# The utility and validity of pain intensity rating scales for use in developing countries

**DOI:** 10.1097/PR9.0000000000000672

**Published:** 2018-08-06

**Authors:** Anupa Pathak, Saurab Sharma, Mark P. Jensen

**Affiliations:** a Department of Physiotherapy, Kathmandu University School of Medical Sciences, Dhulikhel, Nepal; b Centre for Musculoskeletal Outcomes Research, School of Medicine, University of Otago, Dunedin, New Zealand; c Department of Rehabilitation Medicine, University of Washington, Seattle, WA, USA

**Keywords:** Pain intensity, Pain assessment, Culture, Pain measurement, Musculoskeletal pain

## Abstract

**Introduction:**

Pain intensity is the domain most often assessed in pain research. Although the Numerical Rating Scale is recommended for use in western countries, the utility and validity of this scale, relative to others, has not been established in non–western developing countries, such as Nepal.

**Objectives:**

Here, we sought to (1) identify which of 4 commonly used pain scales is most preferred by Nepalese, (2) compare error rates, (3) determine whether preference and error rates are influenced by age or education level, and (4) evaluate construct validity of each scale using factor analysis.

**Methods:**

Two hundred two adults with musculoskeletal pain from Nepal rated their worst and average pain intensity using all 4 scales and selected their most preferred scale.

**Results:**

The results indicate that the Faces Pain Scale-Revised is the most preferred scale, followed by a Verbal Rating Scale. The Numerical Rating Scale and Visual Analogue Scale were both least preferred and had higher rates of incorrect responses, especially among the older participants. However, all the scales demonstrated adequate construct validity as measures of pain intensity among those participants who could accurately use all 4 scales.

**Conclusion:**

The findings indicate that the Faces Pain Scale-Revised should be the first choice for assessing pain intensity in Nepalese adults. Research is needed to determine whether these findings replicate in other non–western and developing countries, to identify the pain intensity measure that would be the best choice for use in cross-cultural pain research.

## 1. Introduction

Pain intensity is a common outcome domain assessed in pain clinical trials^1,14,21,25,31,34,38,45^ and most often targeted in pain treatment.^34,42^ Different domains of pain intensity such as current pain intensity, 24-hour average pain intensity, worst pain, and least pain are assessed regularly in clinical practice and research studies. Although an individual's average pain is arguably the more important outcome domain to target in pain treatment, research comparing average and worst pain ratings indicate that worst pain is more strongly associated with disability.^30^ Thus, both average and worst pain remain important intensity domains to assess, and knowledge regarding the validity and utility of both is important.

Pain intensity is commonly assessed using measures such as the Visual Analogue Scale (VAS), Numerical Rating Scale (NRS), Verbal Rating Scale (VRS), and Faces Pain Scale-Revised (FPS-R).^29,31^ Consensus groups recommend using NRS for clinical research^12^ given its strengths as found in research in samples of individuals from western countries.^4,6–8,15,16,18,31,38,39^ Thus, it might be reasonable to conclude that the NRS should be the measure of choice in most settings.

However, each scale has important strengths and weakness, and no one scale is recommended for use in all situations and with all patient groups.^14,25,31^ Faces scales (ie, pain scales that illustrate different levels of pain intensity through different facial expressions), for example, were developed for use in children and people with low literacy levels. However, there are concerns that such scales may also be influenced by, or assess, emotional reactions in addition to pain intensity.^22,34,35^ Moreover, there are inconsistencies in the way different individuals interpret measurement scales,^13^ and the most useful measure may vary between populations as a function of age, literacy levels, and cultural background.^34,38^ For example, the VRS and the FPS-R (or the scale on which the FPS-R is based, the FPS^24^) are often preferred over other measures,^33,34,43,47^ especially by individuals with lower education levels.^9,21,46^ There is also evidence that the NRS and VRS may not provide reliable measures of pain intensity in individuals from developing countries who have less than 7 years of education.^28^ Although studies report no differences in scale preference based on age,^21,34,43^ the VAS is known to be more difficult to use than other scales,^23^ especially among the elderly^12,34,38^ and individuals with cognitive deficits.^10,21^

Pain perception and expression is influenced by culture and ethnicity as well.^11,32^ Studies performed in samples from the USA and Europe report a higher preference for the NRS,^15,38^ whereas the FPS or the FPS-R tend to be preferred by Turkish, and Chinese individuals.^33,34,47^ In addition, one study of individuals from Ghana found that a colored circle pain scale was preferred over both the FPS and NRS.^2^ Based on these findings, and in light of the fact that most research in this area have been performed in samples from western countries, it seems necessary to evaluate the psychometric properties of pain intensity measures in developing countries, before the NRS can be recommended over other scales for cross-cultural research.

Unlike in western societies where citizens are exposed to a variety of response scales as the part of day-to-day life (eg, online or paper feedback forms for customer feedback in banks and medical facilities), the population in Nepal is rarely exposed to or asked to complete such measures, due in part to the low literacy rates in Nepal.^17^ Although a majority of the population are able to count to 10 and perform simple calculations associated with money handling, patients frequently fail to understand and use pain scales such as NRS in clinical settings.

Given these considerations, the aim of this study was to evaluate the utility and validity of 4 pain intensity measures in a sample of individuals from a non-western and low literacy country (in this case, Nepal). We also sought to understand the role of age and education on the utility and validity of the measures. Based on research findings cited previously, and given the relatively low literacy rates in Nepal, we hypothesized that the study participants would prefer the verbal (VRS) and pictorial (FPS-R) scales over the numerical (NRS) or analogue (VAS) scales. We also hypothesized that while preference rates would not be affected by age, participants with less education would prefer the FPS-R and VRS over the NRS and VAS. Third, we hypothesized there would be more errors in the NRS and VAS, relative to the FPS-R and VRS. Fourth, we hypothesized that older participants would have more incorrect responses to all measures, but error rates will not vary as a function of education level. Finally, we hypothesized that all the scales would demonstrate adequate construct validity, as reflected by large factor loadings on the first factor that emerges from factor analysis of the scale responses.

## 2. Methods

We conducted a cross-sectional study in Nepal, recruiting participants from 3 settings: (1) a tertiary care hospital in Nepal located 30 km from Kathmandu; (2) rural, semiurban, and urban community settings as reflected by 3 districts in Nepal (namely Kavre, Kathmandu, and Lalitpur); and (3) a group home for the elderly located in Kathmandu. The study was approved by the Institutional Review Committee (IRC) at Kathmandu University School of Medical Sciences (KUSMS), Nepal. Data collection was conducted by the first author (A.P.), then a fourth year physiotherapy student at KUSMS, between July 2017 and October 2017, using convenience sampling. This was an independent study that was conducted as a Bachelor of Physiotherapy thesis project by A.P., supervised by the 2 other authors (S.S. and M.P.J.). Written informed consent was obtained from all the participants who could read and write. If participants were unable to read and write, a witness signed the consent form on their behalf.

### 2.1. Participants

We invited individuals with self-reported or clinician-diagnosed musculoskeletal pain to participate in the study. Musculoskeletal pain was defined as pain in any part of the body that potentially originates from the musculoskeletal system, ie, muscles, ligaments, bones, or joints in that region. This excludes pain because of pathologies such as tumors, fractures, infections, and systemic and neurological causes.^26^ Those who expressed an interest in participating in the research were screened for inclusion either using a detailed pain history or their medical diagnosis if it was available. Participants were included if they (1) were 18 years or older; (2) currently had self-reported or clinician diagnosed musculoskeletal pain of any duration; (3) could speak and understand Nepali; and (4) had no motor difficulty of their hands. For self-reported pain, site and quality of pain (using body chart); behaviour of pain; cause of pain; aggravating and relieving factors; and presence of co-morbidities were used to determine whether the participants had musculoskeletal pain or not. We excluded participants with a medically diagnosed history of cognitive impairment and/or visual impairment. A total of 210 participants were screened, of which 202 met the inclusion criteria; 3 were excluded because of lack of fluency in Nepali, 2 because of history of neurological disorder that interfered with participation, and 2 because of recent fracture. Among those included, 151, 25, and 26 participants were recruited from the hospital, community, and the old age home, respectively.

### 2.2. Translation of pain intensity measures into Nepali

During the conception of the study, 3 of the 4 proposed measures were not available in Nepali. Hence, we first translated these scales (FPS-R, VRS, and VAS) into Nepali by adapting standard recommended translation guidelines using forward and backward translation methods.^3^

### 2.3. Measures

#### 2.3.1. Faces Pain Scale-Revised

The FPS-R (2001, International Association for the Study of Pain [IASP]), used with permission from the IASP, is a self-reported pictorial scale that consists of 6 faces showing increasing levels of pain. The respondents are asked to select a face that best represents their level of pain at the time of assessment. Faces from left to right are scored as 0, 2, 4, 6, 8, and 10 by the administrator.^24^ Although FPS-R was originally designed for use in children, it is also commonly used in adult populations, especially in the elderly and those with low literacy.^34,36,46^ The FPS-R was adapted from the original FPS developed by Bieri et al.,^5^ which consisted of 7 faces. The revision of FPS to FPS-R was performed to create a scale that is more compatible with the common 0 to 10 metric score such as those used for the NRS and VAS.^24^

#### 2.3.2. Verbal Rating Scale

The VRS, also sometimes referred to as the verbal descriptor scale, consists of adjectives or phrases that describe increasing intensities of pain. We used the 6-point VRS scale used by Peters et al.,^38^ with the descriptors “no pain,” “very mild,” “mild,” “moderate,” “severe,” and “very severe.” Each descriptor has a number associated with it (eg, “no pain” = 0 and “very severe” = 5). The respondent is asked to select the descriptor or phrase that best represents their pain intensity, and the corresponding number is used as the VRS score.^29^

#### 2.3.3. Numerical Rating Scale

The 11-point NRS consists of numbers between 0 and 10 where 0 indicates “no pain” and 10 indicates “maximum pain.”^40^ The respondent is instructed to identify one number between 0 and 10, which is best representative of their pain intensity. The measure has been shown to be valid and reliable in Nepalese adults with musculoskeletal pain who can count numbers between 0 and 10 with an excellent 2-week test–retest reliability (intraclass correlation coefficient = 0.81).^40^

#### 2.3.4. Visual Analogue Scale

We used a mechanical VAS, which consists of a plastic ruler with a 100-mm line, where the length of the line denotes the severity of pain. We used the same anchors as the NRS for the VAS (ie, 0 mm = “no pain” and 100 mm = “maximum pain”). For the VAS used here, we translated the instructions described by Hawker et al.^20^ The participants were instructed to slide the indicator (a straight blue line that is perpendicular to the 100-mm line) along the length of the line to the point that best represents their pain intensity. Scoring is performed by measuring the length from 0 mm to the respondent's mark.^20^ The VAS can be administered in 3 forms as a graphic scale on paper, using a mechanical ruler or electronically. Both the mechanical VAS and electronic VAS have demonstrated strong associations with the paper VAS.^27^

### 2.4. Procedures

We first asked each participant to rate their worst pain and average pain in the past week using all 4 scales (FPS-R, VRS, VAS, and NRS). Participants were asked to rate both the average and worst pain using one scale before providing a response to the next scale, and the scales were presented on different pages (so they could not easily refer to their previous responses when responding to each scale). Hence, all the participants provided 8 ratings. Scale presentation order was randomized using a Latin square design. All participants were given the instructions for each scale verbally, which were repeated a maximum of 3 times if they were unable to provide a response to the scale based on the instructions provided. If any participant answered incorrectly to any measure, the administrator did not attempt to facilitate a correct response (other than to repeat the instructions, if requested by the participant). After participants had provided the 8 ratings using the 4 scales, they were asked to identify the scale that they found the easiest to understand or use and would prefer to use in the future; they were also allowed to indicate no preference.

Each response was then classified as being either correct or incorrect. A response was recorded as “incorrect” and coded accordingly if any of the following was true: (1) participants were unable to provide a response even after the instructions were repeated 3 times; (2) participants provided a range of pain intensities instead of a single score (eg, “3–5” when asked to indicate their pain intensity on the NRS); (3) if their “worst” pain rating was less than their “average” pain rating for that scale; (4) the participant provided 2 or more responses to a scale (eg, 2 or more distances on the VAS, 2 or more faces on the FPS-R, 2 more numbers for the NRS, etc.); or (5) if they answered beyond the end point of the scale (eg, “11” on the 0–10 NRS).^31,38^

### 2.5. Data analyses

First, we computed descriptive statistics for the demographic characteristics and pain variables to describe the sample. To test the first study hypothesis (that the study participants would prefer the VRS and FPS-R over the NRS and VAS), we performed an omnibus χ^2^ goodness-of-fit analysis comparing the preference rates for the scales. In the event of a significant omnibus test, we planned to then perform a series of χ^2^ tests to evaluate the preference rates between each pair of scales. To test the hypotheses that older participants and participants with lower levels of education would prefer the FPS-R and VRS more than the NRS and VAS, we first classified the participants into groups based on their education and age. We grouped each participant as (1) older (ie, 60 years and older) or (2) younger (ie, 59 years or younger), based on the *Senior Citizen's Act*, 2006, in Nepal, which defines a senior citizen as any citizen of Nepal who is 60 years or older.^37^ Previous studies looking into the effect of age on preference and psychometric properties of pain intensity scales have also used this same cutoff age (60 years) as older population.^37^ With respect to education level, we classified each participant as having (1) more education (completing grade 6 or higher) or (2) less education (completing grade 5 or less). Grade 5 was chosen as a cutoff because grades 1 through 5 are classified as primary education in Nepal, which has a national goal for all citizens to successfully complete at least primary education. We then evaluated preference rates for the scales for each age and educational level group separately using 4 omnibus χ^2^ analyses. In the event of a significant omnibus test, we planned to perform a series of pairwise χ^2^ analyses or Fisher's exact test (if any of the cell counts were less than 5) to identify which scales were preferred over the others in each of the 4 groups. To address the third study hypothesis that there would be a greater number of incorrect responses with scales requiring more abstract thinking (ie, the NRS and the VAS) than those that require less abstract thinking (ie, the FPS-R and VRS), we compared the rates of correct vs incorrect responses between each pair of scales using the McNemar test. To test the fourth study hypothesis regarding error rates as function of age and education level, we performed a series of χ^2^ tests to compare the error rates of individual scales between older and younger participants as well as between participants with more education and less education. We also classified the types of errors that participants made to each measure for descriptive purposes. To address the final study hypothesis, 2 factor analyses were performed, using principal axis rotation: 1 for the average pain and the other for the worst pain ratings. Only the participants who responded accurately to all 4 scales were included in the principal axis factor analyses. Based on previous research that consistently finds that a single factor emerges when different measures of pain intensity are entered in a factor analysis,^15,23,31,38^ we hypothesized that a single factor would emerge from these analyses as evidenced by a high eigenvalue for the first factor and lower eigenvalues (ie, <1.0) for the remaining factors. We then planned to examine the magnitude of the loading of each scale on this factor as an indication of each scale's construct validity.^31^ All data analyses were performed using the Statistical Package for Social Sciences (SPSS) version 16.

## 3. Results

### 3.1. Description of sample

Demographic characteristics of the study sample are presented in Table 1. As can be seen, 48% (n = 97) of the participants were older adults and the mean age of the participants was 54 years (SD 19 years, range = 18–90 years). Women comprised 57% (n = 115) of the sample. Over half of the sample (60%, n = 122) had only completed 5 years of education or fewer. A majority (66%, n = 81) of those who were less educated belonged to the older age category (60 years or older). About one-third of the participants (29%, n = 59) reported having more than one pain problem. The most commonly reported pain locations were knee (46%, n = 93), low back (38%, n = 76), and shoulder (15%, n = 31). The median duration of pain was 6 months (range 2 days to 35 years), and 60% (n = 122) of the participants had persistent pain (ie, pain for more than 3 months) as per the definition of IASP.^44^

**Table 1:**
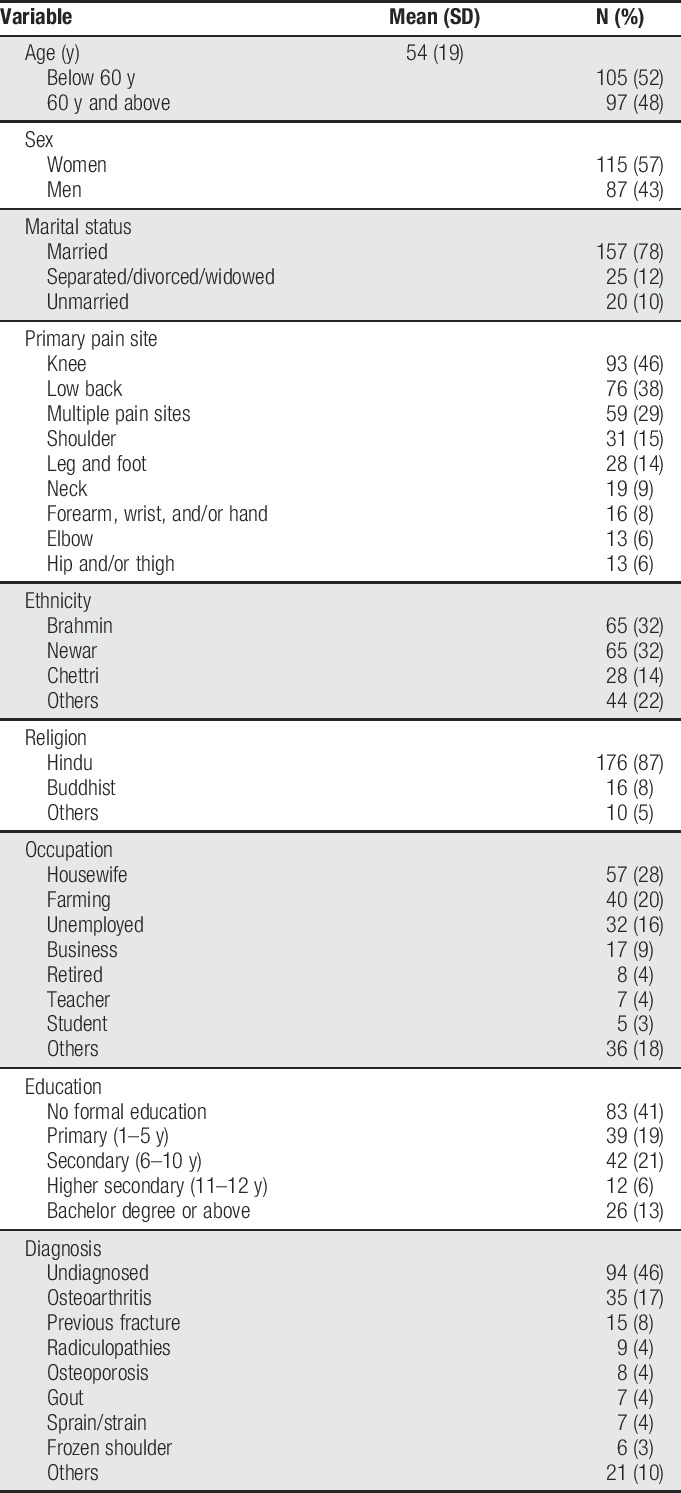
Participant descriptive information.

### 3.2. Scale preferences

The omnibus χ^2^ test for goodness-of-fit (hypothesizing equal proportions, ie, 20% preference of each scale) indicated a statistically significant difference in scale preferences for the sample as a whole (χ^2^(4) = 42.11, *P* < 0.001). Pairwise comparisons between each scale with respect to preference, again hypothesizing equal proportions (ie, 50% each), indicated significant differences between the FPS-R and all the other scales (χ^2^(1) range = 11.90–27.04, all *P*s ≤ 0.001). However, no significant differences were found for any other pairs (χ^2^(1) range = 0.67–5.57, *P*s range = 0.059–0.279). Overall, the preferred scale was the FPS-R (38%, n = 76), followed by the VRS (19%, n = 39), VAS (15%, n = 30), and NRS (12%, n = 24). Thirty-three participants (16%) did not prefer any single scale over the others.

### 3.3. Scale preference as a function of age and education

A majority of the older (34%, n = 33), as well as the younger (41%, n = 44) participants, preferred the FPS-R, whereas the NRS was least preferred by the older participants (7%, n = 7). The results of the omnibus χ^2^ test indicated that differences in rates of preference of the 4 scales were significant for both younger and older participants (*P* < 0.001; Table 2). Follow-up χ^2^ tests examining the preference rates between each scale (none of the cells had less than 5 participants) revealed that the FPS-R is preferred over all of the other scales by both younger and older participants. No significant differences were found in the preference rates for the VRS, VAS, and NRS in the younger group, and the VRS and VAS in the older group (Table 2). The FPS-R was also the preferred scale in both education groups (less educated, 43%, n = 52; more educated, 31%, n = 24). However, the higher preference rate for the FPS-R was only significantly greater among those with less education (Table 2). For the more educated group, no significant differences were found between the preference rate of the FPS-R, compared with the other 3 scales.

**Table 2:**
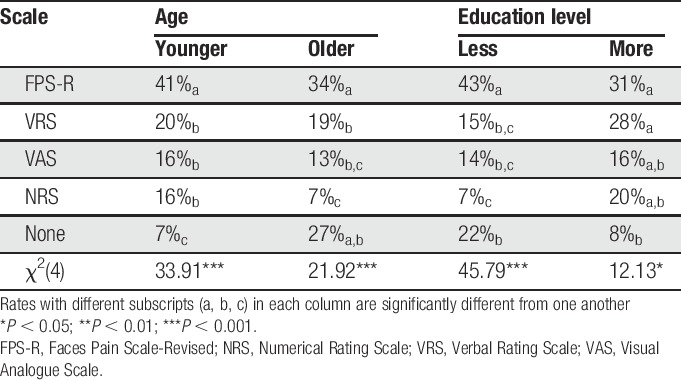
Scale preference rates as a function of age and education.

### 3.4. Rates of incorrect responses

Of 202 participants, 9% (n = 18) made errors in all 4 scales. The results of the series of McNemar tests indicated significant between-group differences in incorrect response rates for all pairs of ratings, except for those between the FPS-R and the VRS (Table 3). As can be seen, the highest rate of incorrect responses was observed for the NRS (64%, n = 129) followed by the VAS (33%, n = 66) and then the VRS (24%, n = 49). The least number of incorrect response was observed with FPS-R (18%, n = 37).

**Table 3:**
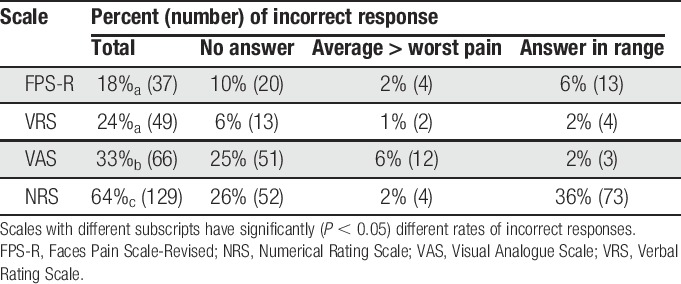
Comparison of error rates among the scales with most common errors.

The most common error in responding to the NRS was providing a range of pain intensities (eg, “1–2”) rather than a single number; 36% of the participants responding to the NRS provided this incorrect response. Twenty-six percent of the participants were unable to rate their pain intensity at all using the VAS, and the same percentage (26%) was unable to provide a response to the NRS. The least number of nonresponses was seen for the VRS, with only 6% of the participants failing to provide any response at all to this measure. The most common error for the VRS (15%) was for participants using different descriptors or phrases than those provided by the VRS that was offered.

### 3.5. Incorrect responding rates as a function of age and education level

The rates of incorrect responding to the 4 scales as a function of age and education level are presented in Table 4. As can be seen, χ^2^ tests of independence evidenced significant differences in the error rate for all scales as a function of both age and education level, ie, older participants and less educated participants had higher incorrect response rates across all scales.

**Table 4:**
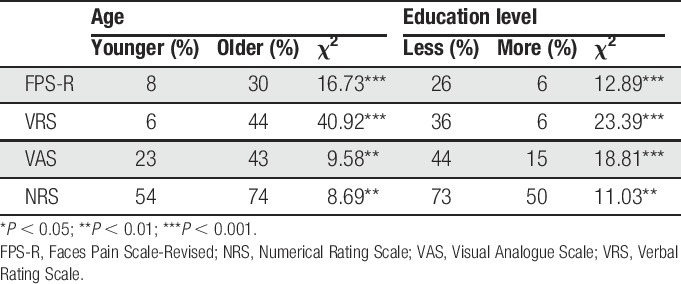
Percentages of incorrect responding rates as a function of age and education level.

In terms of types of error, 44% and 41% of the older and less educated participants were unable to provide any pain rating at all with the NRS, respectively. By contrast, the most common error in NRS use among the younger participants (43%) and those with more education (46%) was answering with a range of numbers (eg, “2–4”). A similar finding was seen in the VAS, where 40% of older and 35% less educated participants were unable to rate their pain using the VAS.

### 3.6. Construct validity

Only 34% (n *=* 68) of the participants correctly reported their average pain in response to all 4 scales, and 43% (n = 86) participants correctly reported their maximum pain with all 4 scales. The results of the principal axis factor analyses for these ratings are presented in Table 5 and provided strong support for a single factor for both average pain (eigenvalues = 2.70, 0.60, 0.43, and 0.28) and maximum pain (eigenvalues = 2.93, 0.43, 0.34, and 0.30). Among the scales, the NRS demonstrated the highest factor loading for both maximum pain (0.84) and average pain (0.88). However, even the least of the loadings (FPS-R for maximum pain, loading = 0.75; VAS for average pain, loading = 0.69) were high.

**Table 5:**
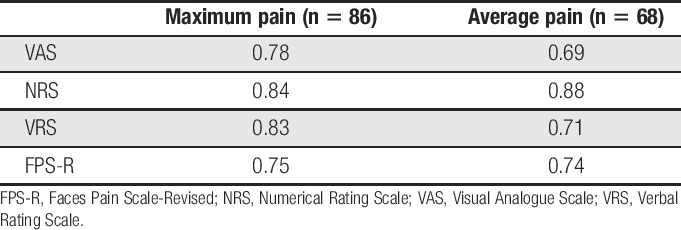
Factor loadings on the first factor of the principal axis factor analyses.

## 4. Discussion

Perhaps the most important finding from this study is the large number of incorrect response seen in NRS in this sample of individuals from a nonwestern country with low literacy rates. Also, and as hypothesized, we found that the rates of incorrect responding varied as a function of age, and that the FPS-R and VRS were preferred over the VAS and NRS. All scales evidenced validity based on factor analysis, at least among those participants who were able to provide a response to all of the scales. These findings have important implications for selection of pain intensity measures in cross-cultural pain research and for use in developing countries that have low literacy rates.

### 4.1. Scale preference

As hypothesized, the non-numerical scales (FPS-R and VRS) were preferred over the VAS and NRS. This finding is in line with the findings from China and Turkey, where the FPS and FPS-R were also found to be preferred over other scales.^33,34,47,49^ These findings contrast with those studies using samples from western countries, which tend to find higher preference rates for the NRS over the VRS (although several of these studies did not compare these 2 scales with the FPS or FPS-R).^15,31,38,48^

There may be important cultural reasons why the FPS-R and VRS are preferred by individuals in our sample as well as those from China.^34^ In Nepalese communities, it is uncommon to use numerical scales, such as the NRS, in daily life. In addition, descriptive terms such as a “handful of rice” and “forearm's length” are generally used to describe quantities and lengths. Although numbers are also sometimes used in daily life, abstract quantities such as magnitudes are generally described using words rather than numbers. Scales such as the VAS or the NRS, on the other hand, require converting the magnitude of a sensation into a length on a line or number. To reliably make such conversions, one may need previous experience with such transformations in other contexts. Therefore, the lower preference for VAS and NRS in our sample is due to lack of experience in using scales, rather than an inability to use numbers.

We had hypothesized that the FPS-R and VRS would be preferred over the other scales by less educated participants. In support of this hypothesis, a greater preference for the FPS-R (43%) over the other scales (15%, 14%, and 7%) was found in the less educated group. Also, and as expected, we did not find any between-group differences in preference rates between younger and older participants. These results are consistent with previous studies, which indicate high preference rates for verbal descriptor and FPSs, irrespective of age.^21,34,43,47^ Clark et al., as well as Sayin et al., also reported that participants with more education preferred the VAS.^9,47^ Overall, the findings indicate that faces scales (such as the FPS-R) and VRS tend to be the preferred scales in nonwestern countries.

### 4.2. Rates of incorrect response

The highest rate of incorrect response in our study was seen with the NRS (64%) followed by the VAS (33%). A similar pattern of findings was reported in samples of patients from China and Canada.^15,34^ However, the error rates for all 4 measures tended to be higher in our sample relative to other studies. For example, 19% of our participants made errors on all 4 scales, which are very high compared with an U.S. sample, where only 1% participants made errors in all of the 3 scales examined.^21^ The differences could be due to the relatively low rate of literacy in our sample, cultural differences in how magnitude is usually communicated, or a combination of these.

As hypothesized, we observed higher error rates among the older participants across all 4 scales. The VAS and NRS were particularly problematic for the older and less educated participants, many of whom were unable to use these scales even after the instructions were repeated. Previous studies comparing the use of scales in younger and older participants have also reported increased errors among older individuals across pain intensity measures, with particular difficulty in using the VAS.^12,15,23,34,38^ Also as hypothesized, no significant association was found between education level and error rates in our study, which is consistent with previous research in other countries.^15,21,38^ Overall, the findings suggest that the FPS-R may be more useful than the VAS, NRS, or VRS in research studies that include elderly individuals.

### 4.3. Scale validity

Consistent with the findings from previous studies,^31,34,38^ among the subsample of individuals who were able to provide a response to all 4 scales, all the scales demonstrated adequate construct validity as evidenced by strong loadings on the single factor that emerged from a factor analysis of these scales. Also, the NRS demonstrated the highest loading on the factors representing both average and worst pain intensity. This generally consistent finding with respect to the NRS may be due to the possibility that the 0 to 10 NRS provides enough response options to allow for adequate precision in rating intensity (which has been shown to have roughly 21 just noticeable differences between no pain and extreme pain),^19^ while at the same time, the 0 to 10 NRS provides the respondent with some limits in the number of responses (ie, unlike the VAS, which provides essentially an infinite number of possible ratings along a continuum); limits which may help to minimize the complexity of the rating task.^29,34^ However, it is important to note that the findings from factor analyses do not provide complete information about a measure's validity; research examining other validity criteria for the measures of pain intensity evaluated in this study is needed to more fully understand the psychometric strengths and weaknesses of these measures in individuals from Nepal.

### 4.4. Study limitations

The study has a number of limitations which should be considered when interpreting the results. Perhaps the most important limitation is the lack of previous research validating 3 of the translated pain intensity scales used in the study (ie, the VAS, FPS-R, and VRS). For example, the descriptors representing different levels of pain intensity in the VRS were translated directly from a standard VRS measure that was developed in another country and not selected based on the words used by Nepalese to describe different pain magnitudes. Thus, the descriptors representing different levels of pain intensity in the VRS used here may not have been the most familiar adjectives that Nepalese use to describe increasing intensities of pain. This possibility is supported by the finding that 15% of the participants spontaneously mentioned different adjectives (than those on the VRS used) to describe their pain intensities. Work to identify the most common words that Nepalese use to describe the magnitude of felt pain would allow us to determine whether a more useful VRS specific to Nepalese populations could be developed. Second, we did not take into consideration previous exposure to the scales. Previous studies have reported decreased error rates after repeated exposure to pain intensity scales.^15,34^ Thus, the error rates reported here might have been lower had we recruited participants who had more experience with these scales. Also, and related to this issue, it is possible that the error rates might have been lower had we included procedures for training the participants in the use of the measures.^41^ Third, we did not assess the cognitive status of the research participants. Doing so could have helped us to understand the extent to which the higher error rates among the older participants were due to age-related cognitive dysfunction or other factors, such as age cohort effects (ie, younger individuals may have more exposure and experience with rating their experience using numbers or line lengths). Fourth, we did not consider any bias that may have resulted as a result of the ethnicity and sex of the researcher administering the scales. It is possible, for example, that different findings might have emerged had the interviewer been from different ethnic group or gender. Finally, the study sample consisted of individuals with musculoskeletal pain. Thus, the extent to which the findings generalize to samples of individuals from Nepal with chronic neuropathic pain is not known. Replication of the study in these additional samples is warranted. In addition, future researchers should also examine the role of chronicity, researcher sex, and researcher ethnicity on preference of pain intensity measures.

### 4.5. Summary and conclusions

Based on the current findings, and in light of the findings from other studies, it would seem that the most useful measure of pain intensity in Nepal—and perhaps in other nonwestern countries with low literacy rates—may be the FPS-R, followed by the VRS. Although support for the validity of all 4 scales was found among the subsample of participants who provided ratings on all 4 scales, use of the NRS or VAS in a sample of individuals in nonwestern or developing countries with low literacy rates may result in unacceptably high rates of missing data.

In addition, although it might be reasonable for researchers in western countries to use the NRS as their primary measure (based on consensus recommendations^12^), they should consider also using and reporting the results from measures such as the FPS-R or VRS because (1) adding one or both of these measures would not substantially increase assessment burden and (2) reporting results using these additional scales (as secondary outcomes) would allow for greater opportunities for between-study and cross-cultural comparisons of study findings. Given the high preference of FPS-R observed with this study and previous studies, as well as previous research, suggesting that the FPS-R might be biased or influenced by the emotional components of pain, there is also a need for further research to evaluate the validity of the FPS-R, in particular, in more samples of individuals with pain.

## Disclosures

The authors declare no conflict of interest. The International Association for the Study of Pain (IASP) did not have any influence on the analysis or reporting of the study findings.

Findings of the study were presented at the World Confederation for Physical Therapy (WCPT) Conference, July 3, 2017 in Cape Town by the first author during poster presentation.
